# Alfalfa Mosaic Virus and White Clover Mosaic Virus Combined Infection Leads to Chloroplast Destruction and Alterations in Photosynthetic Characteristics of *Nicotiana benthamiana*

**DOI:** 10.3390/v16081255

**Published:** 2024-08-05

**Authors:** Yinge Chen, Qiaolan Liang, Liexin Wei, Xin Zhou

**Affiliations:** Biocontrol Engineering Laboratory of Crop Diseases and Pests, College of Plant Protection, Gansu Agricultural University, Lanzhou 730070, China

**Keywords:** *Nicotiana benthamiana*, alfalfa mosaic virus, white clover mosaic virus, virus combined infection, chloroplast, photosynthetic characteristics

## Abstract

Alfalfa mosaic virus (AMV) is one of the most widely distributed viruses; it often exhibits combined infection with white clover mosaic virus (WCMV). Even so, little is known about the effects of co-infection with AMV and WCMV on plants. To determine whether there is a synergistic effect of AMV and WCMV co-infection, virus co-infection was studied by electron microscopy, the double-antibody sandwich enzyme-linked immunosorbent assay (DAS-ELISA), and real-time fluorescence quantitative PCR (RT-qPCR) of AMV and WCMV co-infection in *Nicotiana benthamiana*. Meanwhile, measurements were carried out on the photosynthetic pigments, photosynthetic gas exchange parameters, and chlorophyll fluorescence parameters. The results showed that the most severe disease development was induced by AMV and WCMV co-infection, and the disease grade was scale 7. *N. benthamiana* leaves induced mottled yellow-green alternating patterns, leaf wrinkling, and chlorosis, and chloroplasts were observed to be on the verge of disintegration. The relative accumulation of AMV CP and WCMV CP was significantly increased by 15.44-fold and 10.04-fold upon co-infection compared to that with AMV and WCMV single infection at 21 dpi. In addition, chlorophyll a, chlorophyll b, total chlorophyll, the net photosynthetic rate, the water use efficiency, the apparent electron transport rate, the PSII maximum photochemical efficiency, the actual photochemical quantum yield, and photochemical quenching were significantly reduced in leaves co-infected with AMV and WCMV compared to AMV- or WCMV-infected leaves and CK. On the contrary, the carotenoid content, transpiration rate, stomatal conductance, intercellular CO_2_ concentration, minimal fluorescence value, and non-photochemical quenching were significantly increased. These findings suggest that there was a synergistic effect between AMV and WCMV, and AMV and WCMV co-infection severely impacted the normal function of photosynthesis in *N. benthamiana*.

## 1. Introduction

Plant diseases caused by pathogens decrease crop yields and quality in the world, about half of which are caused by viruses [1,2]. Thus, the timely assessment of viral infection and spread in host plants is the prerequisite for an efficient crop protection plan, either in the field or in the greenhouse [3,4]. However, with the further study of viruses, the phenomenon of co-infection with multiple viruses has been found [5,6]. This co-infection not only aggravated the occurrence of the disease but also caused more serious disease symptoms such as chlorosis, wrinkling, and the mottling of plants, resulting in a reduced chloroplast pigment content, destruction of chloroplast structure, changes in their structure and function, a significantly decreased photosynthesis rate, and a greatly reduced yield and quality of the crop [7,8,9].

Alfalfa mosaic virus (AMV)—also known as *Alfamovirus AMV*—which is a member of the genus *Alfamovirus* in the family Bromoviridae [10], and white clover mosaic virus (WCMV/WCIMV), defined in this paper as WCMV and also known as *Potexvirus trifolii*—which is a member of the genus *Potexvirus* in the family Alphaflexiviridae [11]—exhibit naturally combined infections on a wide range of plant species, including forages such as alfalfa and clover, and cash crops such as *Nicotiana benthamiana*, *Solanum tuberosum*, and *Capsicum annuum*, resulting in serious economic losses [12,13,14,15,16]. In our preliminary studies on naturally infected alfalfa, we detected a high rate of co-infection with two viruses, AMV and WCMV, which resulted in the yellowing and shrinking of alfalfa leaves. The chlorophyll content and soluble protein content were lower than those of AMV and WCMV single infections [17]. Studies have also revealed that the co-infection with AMV and WCMV aggravated the symptoms of the disease in *N. benthamiana*, such as mottled, crumpled, and rebloomed leaves that appeared yellowish-green or in shades of green [12]. When the mixture ratio of AMV and WCMV was 3:1, the most severe symptoms were observed in the co-infection, with an average disease index of 80.12, which was increased by 22.36% and 45.28%, respectively, when compared with AMV and WCMV single infections [16]. It has also been reported that when the virus infects plants, chloroplasts are the main target for its pathogenicity, replication, and multiplication, resulting in the destruction of chloroplast structure and a decrease in chlorophyll content, with the host showing symptoms such as leaf mosaic and yellowing, which affect photosynthesis [9,18]. However, the effects of AMV and WCMV co-infection on the destruction of chloroplast structure, pigment content, and photosynthetic parameters of *N. benthamiana* have not been reported.

Thus, in this study, we used AMV and WCMV as research subjects and *N. benthamiana* as treatment hosts. After inoculation with a mixed viral solution of AMV and WCMV (3:1) and a single inoculation with AMV and WCMV, respectively, symptoms of the disease and the morphological structure of chloroplasts were observed; AMV and WCMV concentrations were assayed; and the relative accumulation of viral capsid proteins and photosynthetic pigment content and the changes in photosynthetic characteristics were determined. The objective of this study was to determine the symptoms and virus accumulation caused by AMV and WCMV co-infections. The effects of AMV, WCMV, and host interaction on chloroplast ultrastructure and photosynthetic characteristics were investigated, and the results will provide a theoretical basis for revealing the pathogenic mechanism of AMV and WCMV combined infection on plants and formulating new strategies for virus prevention and control.

## 2. Materials and Methods

### 2.1. Plant and Virus Materials

*N. benthamiana* plants were soil-grown in an artificial intelligence climate chamber (Hangzhou Qisheng Electronic Technology Co., Ltd., Hangzhou, China) under a 16 h light/8 h dark cycle at 25 °C with a relative humidity of 50%. AMV- and WCMV-purified viruses were stored in a −80 °C refrigerator (Gansu Agricultural University, Lanzhou, China) at a concentration of 300 pg·mL^−1^.

### 2.2. Virus Inoculation

Leaves of *N. benthamiana* plant seedlings with a mixture inoculated with AMV and WCMV (3:1) were used as treatments, AMV and WCMV were single-inoculated as positive controls, and only 0.02 moL·L^−1^ of phosphate-buffered solution (PBS, pH 7.0) was used as the blank control (CK) through the friction inoculation method [17]. Briefly, there were at least three biological replicates of each treatment, and for each replicate, at least 10 seedlings. The plants were labeled and placed in an artificial intelligence climate chamber to observe the symptoms of disease day by day. Samples were collected at 9 days post inoculation (dpi), 15 dpi, 21 dpi, and 27 dpi for the following experimental observation and determination. The scale for rating the disease of *N. benthamiana* was as follows: scale 0, asymptomatic; scale 1, leaf shrinkage; scale 3, slight mosaic and wrinkled; scale 5, moderate mosaic or chlorosis; and scale 7, severe mosaic or necrosis.

### 2.3. Chloroplast Ultrastructure Observations

The treated and control leaves of *N. benthamiana* were collected 21 dpi. Sample treatment for chloroplast ultrastructure observation was as described previously with slight modifications [19]. The processed samples were placed under a JEM1230 LV (Olympus, Osaka, Osaka Prefecture, Japan) for observation and photography.

### 2.4. RT-qPCR Detection

The *N. benthamiana* leaves with AMV and WCMV were analyzed by conventional RT-qPCR. Total RNA was extracted from *N. benthamiana* with TRIzol reagent (Tiangen Company, Beijing, China) according to the manufacturer’s instructions. Next, the extracted RNA was reverse-transcribed to cDNA using the One-Step cDNA kit (Tiangen Company, Beijing, China) in a 20 μL volume, containing 4 μL of 5× FastKing-RT SuperMin, 1 μL of total RNA, and 15 μL of RNase-free water at 42 °C for 15 min and 95 °C for 3 min. Subsequently, quantitative analysis of the AMV and WCMV coat protein (CP) genes was performed by RT-qPCR amplification using the viral cDNA as a template (Tiangen Company, Beijing, China).

The specific primer pairs were AMV-F (5′-GCATCCCTAGGGGCATTCATGCA-3′) and AMV-R (5′-ATCATTGATCGGTAATGGGCCGTT-3′) for AMV CP, and WCMV-F (5′-AAACTCGAGCATGGACTTCACTACTTTA-3′) and WCMV-R (5′-CAGGTACCCTGAA ATTTTATTAAACAGAAAGCACACAC-3′) for the WCMV CP. The 25S ribosomal RNA gene and the β-Actin gene were selected as internal references according to the literature. The specific primer pair was 25S rRNA-F (5′-AAGGCCGAAGAGGAGAAAGG T-3′) and 25S rRNA-R (5′-CGTCCCTTAGGATCGGCTTAC-3′) for the 25S rRNA gene, and β-Actin-F (5′-GAGCACCCTGTTCTTCTGACTG-3′) and β-Actin-R (5′-GAGAAAGAACAGCCTG AATTGC-3′) for the β-Actin gene were selected as the internal reference. These primers were designed using primer software (Oligo Primer Analysis Software v.6.0) based on the coat protein genes of AMV and genomic sequences of WCMV reported in NCBI and synthesized by Shanghai Biological Engineering Company (Shanghai, China).

RT-qPCR was performed in a 20 μL volume, containing 10 μL of Fast Real qPCR Per Mix, 6.5 μL of RNase-free water, 1 μL of upstream and downstream primers (180 nM), 1 μL of cDNA template (100 ng), and 0.5 μL of ROX Reference Dye. The reaction conditions were as follows: pre-denaturation at 95 °C for 15 min, denaturation at 95 °C for 10 s, annealing at 60 °C for 20 s, and extension at 72 °C for 32 s for 40 cycles. Finally, the melting curve analysis was performed at 60 to 95 °C per 0.6 °C increment. Quantitative real-time RT-qPCR analysis was performed on an ABI7300HT Sequence Detection System with Cham QTM SYBR qPCR Master Mix (Applied Biosystems, Foster, CA, USA).

### 2.5. Production and Quantitative Analysis of Standard Curves of Target Gene and Internal Gene

cDNA from the AMV and WCMV co-infection of *N. benthamiana* was used as a standard [20], and it was subjected to 5× gradient dilutions at concentrations of 10 ng·μL^−1^, 2 ng·μL^−1^, 0.4 ng·μL^−1^, 0.080 ng·μL^−1^, and 0.016 ng·μL^−1^, respectively. The primer sequences for amplification, the reaction system, and the amplification procedure are shown in the previous section, and finally, the standard curves for qPCR of the target and internal reference genes were made. The amplification efficiencies (*E*) of AMV CP, WCMV CP, 25S rRNA, and β-Actin genes were calculated, and the relative accumulation of AMV CP and WCMV CP genes was calculated using the 2^−ΔΔCt^ method.

$$E=10^{-1/K}-1$$

where *E* represents the amplification efficiency and *K* represents the slope of the standard curve.

### 2.6. Chlorophyll Extraction and Photosynthetic Pigment Measurement

The photosynthetic pigments were extracted from diseased *N. benthamiana* leaves. Briefly, the veins were removed from each leaf sample. The leaves were homogenized in 95% ethanol with a small amount of CaCO_3_, and the homogenate was centrifuged at 5000 rpm for 5 min (Eppendorf 5430R, Beijing, China) [21]. The absorbance of the supernatant was recorded at wavelengths of 665 nm, 649 nm, and 470 nm (Unicam UV 9000S, Thermo Spectronic, Shanghai, China). The contents of Ch1_a_ and Ch1_b_ and the sum of carotenoids (C_ar_) were calculated using the following equations.

$${Ch1}_{a}=13.95\times A_{665}-6.88\times A_{649}$$

$${Ch1}_{b}=24.96\times A_{649}-7.32\times A_{665}$$

$$\begin{array}{l} T-Ch1={Ch1}_{a}+{Ch1}_{b}\\ C_{ar}=\left(1000\times A_{470}-2.05\times {Ch1}_{a}-114.8\times {Ch1}_{b}\right)/245 \end{array}$$

where Ch1_a_ and Ch1_b_ represent the concentrations of chlorophyll a and chlorophyll b (mg·L^−1^), respectively; T-Ch1 represents the concentration of total chlorophyll (mg·L^−1^); C_ar_ represents the concentration of carotenoid (mg·L^−1^); and A_665_, A_649_, and A_470_ denote the absorbance values of the extracting solution at wavelengths of 665 nm, 649 nm, and 470 nm, respectively.

### 2.7. Photosynthetic Gas Exchange Measurements

Net photosynthetic rate (*P*_n_), intercellular CO_2_ concentration (*C*_i_), transpiration rate (*T*_r_), and stomatal conductance (*G*_s_) in *N. benthamiana* leaves were quantified during sunny mornings from 9:30 to 12:00 on different days after inoculation with AMV and WCMV combined. The gas exchange measurement system gas analyzer GFS-3000 (WALZ, Effeltrich, Germany) is equipped with a standard 8 cm^2^ leaf chamber [22]. The measuring conditions consisted of a light intensity of 150 μmol·m^−2^·s^−1^ and a CO_2_ concentration of 360 μmol·mol^−1^. The air temperature was set at 25 °C and the relative humidity at 48%. The duration of the measurements at each light intensity was 2 min in order to obtain a steady state. Each treatment was replicated three times. All data were based on the average measurements performed on one upper, middle, and lower leaf per replicate [23].

$$WUE=P_{n}/T_{r}$$

### 2.8. Chlorophyll Fluorescence Parameters Measurement

For the PSII quantum yield in the light-adapted state or effective PSII quantum yield (Φ_PSII_), PSII maximum photon yield (*F_v_*/*F_m_*), minimal fluorescence value (*F*_0_), fluorescence (*F_m_*), photochemical quenching coefficient (*q*P), and non-photochemical quenching coefficient (*q*N), a variable chlorophyll fluorescence imaging system (Imaging PAM-MIN, WALZ, Effeltrich, Germany) was used [24]. *F*_0_ was measured under a light intensity of ≤0.5 μmol·m^−2^·s^−1^. Next, *F_m_* was measured using a saturating pulse (2800 μmol·m^−2^·s^−1^). Subsequently, the steady-state fluorescence values (*F_t_*) were measured after exposing the plants to actinic light (600 μmol·m^−2^·s^−1^) for five minutes and then opening a saturating pulse every twenty seconds to measure the adapted maximum fluorescence values ($F_{m}^{\prime }$).

$$F_{v}/F_{m}=\left(F_{m}-F_{0}\right)/F_{m}$$

$$\Phi_{PSII}=\left(F_{m}^{\prime }-F_{t}\right)/F_{m}^{\prime }$$

$$qP=\left(F_{m}^{\prime }-F_{t}\right)/\left(F_{m}^{\prime }-F_{0}\right)$$

$$qN=\left(F_{m}-F_{m}^{\prime }\right)/\left(F_{m}-F_{0}\right)$$

### 2.9. Data Analysis

All statistical analyses were performed using SPSS 21.0. Differences were analyzed using multiple analyses of variance with Duncan’s significant difference tests. A *p* value ≤ 0.05 was considered statistically significant. The data are presented as the mean ± standard error (SE). By comparing the threshold cycle (Ct) values, the relative accumulation of the AMV CP and WCMV CP was analyzed using the comparative 2^−ΔΔCt^ method [25]. Excel 2023 was used to plot the standard curve and every graph.

## 3. Results

### 3.1. Diseased Symptoms and Chloroplast Ultrastructural Changes of N. benthamiana After AMV and WCMV Co-Infection

**Figure 1:**
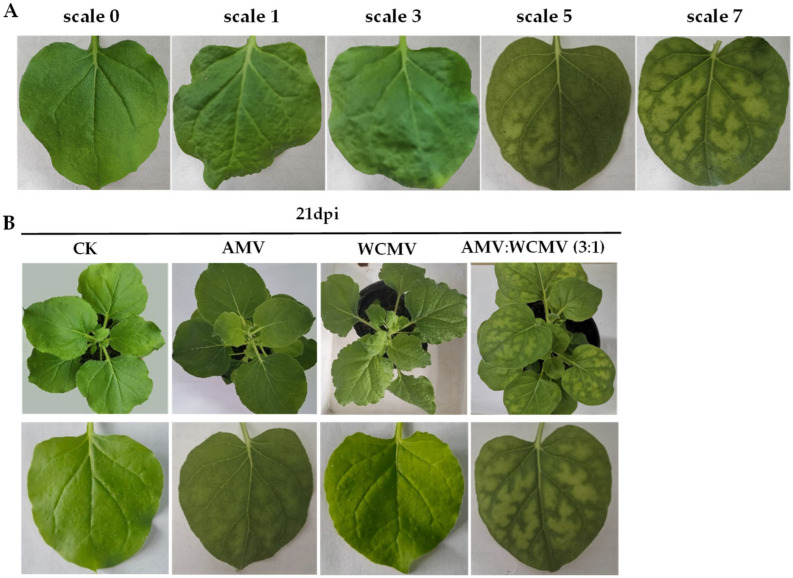
Symptoms of *N. benthamiana* after AMV and WCMV co-infection. (**A**) The rating of disease symptoms of *N. benthamiana* is as follows: scale 0, asymptomatic; scale 1, leaf shrinkage; scale 3, slight mosaic and wrinkled; scale 5, moderate mosaic; and scale 7, severe mosaic. (**B**) Symptoms of *N. benthamiana* after AMV and WCMV co-infection and AMV and WCMV single infection. (CK) Whole plant and simple leaf of healthy *N. benthamiana*. (AMV) AMV infection of whole plant and single leaf of *N. benthamiana*. (WCMV) WCMV infection of whole plant and single leaf of *N. benthamiana*. (AMV:WCMV 3:1) AMV and WCMV co-infection of whole plant and single leaf of *N. benthamiana*. Lower panels are images of upper leaves.

**Figure 2:**
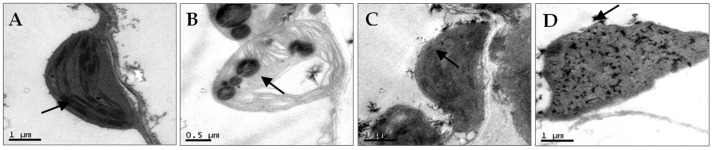
Chloroplast ultrastructural changes of *N. benthamiana* after AMV and WCMV co-infection (21 dpi). (**A**) Image showing the chloroplast structure from CK; the arrow points to a normal granum (magnification, 25,000×). (**B**) The chloroplasts of AMV-infected cells had swollen lipid pellets; the arrow points to starch particles were larger (magnification 40,000×). (**C**) WCMV-infected cells showed abnormal chloroplast structure with no clear grana definition; the arrow points to chloroplast grana were diffuse (magnification, 25,000×). (**D**) Chloroplast inclusions of dissolved AMV- and WCMV-co-infected cells; the chloroplast was on the verge of disintegration; the arrow points to the cell wall showed wavy deformation (magnification, 25,000×).

*N. benthamiana* leaves co-infected with AMV and WCMV exhibited more pronounced disease symptoms than those infected with single AMV or WCMV, and the disease grade reached scale 7 (Figure 1A). The manifested symptoms on co-infected plants included mottle mosaic, crumpling, chlorosis, and deformity (Figure 1B). Meanwhile, the chloroplast was on the verge of disintegration, the cell wall showed wavy deformation, and the periplasm was completely destroyed by *N. benthamiana* leaves co-infected with AMV and WCMV (Figure 2D). Chloroplasts exhibited morphological enlargement with AMV single infection, starch particles were larger than those of plants without inoculation (Figure 2B), and chloroplast grana were diffuse with WCMV single infection (Figure 2C).

### 3.2. Production of Standard Curves and Relative Accumulation of AMV CP and WCMV CP after AMV and WCMV Co-Infection

**Figure 3:**
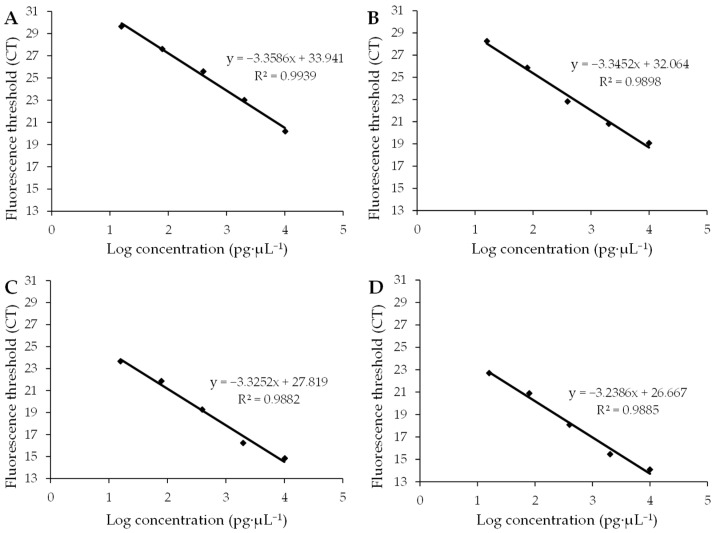
Standard curves of the target gene and internal gene. (**A**) Standard curves of the target gene AMV CP show that the amplification efficiency was 0.9849 with an average standard error of 0.2082. (**B**) Standard curves of the target gene WCMV CP show that the amplification efficiency was 0.9904 with an average standard error of 0.0992. (**C**) Standard curves of the internal gene 25S rRNA show that the amplification efficiency was 0.9986 with an average standard error of 0.0779. (**D**) Standard curves of the internal gene β-Actin show that the amplification efficiency was 1.0360 with an average standard error of 0.1325. R^2^ is a statistic used to measure the degree to which the independent variable explains the change in the dependent variable. When R^2^ approaches 1, it indicates that the independent variable can explain most of the change in the dependent variable’s ability; therefore, the regression equation fits the data well. And when R^2^ approaches 0, the explanatory power of the independent variable compared to the dependent variable is weak, and the regression equation fits the data to a poor degree.

Four standard curves and amplification efficiencies were obtained by plotting standard curves with the Ct value as the vertical coordinate and the logarithmic value of cDNA concentration as the horizontal coordinate (Figure 3). The R^2^ values of the standard curves for the target genes AMV CP and WCMV CP and the internal reference genes 25S rRNA and β-Actin were 0.9939, 0.9898, 0.9882, and 0.9885, respectively, and the amplification efficiencies ranged from 98% to 104% (Table 1). This shows that the linear correlation of the standard curve was good and the amplification efficiency was in the normal range, which is suitable for use in fluorescence quantitative PCR analysis.

**Figure 4:**
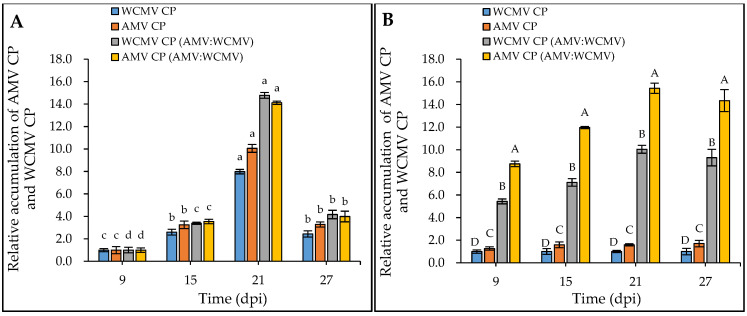
Relative accumulation of AMV CP and WCMV CP in *N. benthamiana* after AMV and WCMV co-infection. WCMV CP, relative accumulation of WCMV CP of WCMV single infection. AMV CP, relative accumulation of AMV CP of AMV single infection. WCMV CP (AMV:WCMV), relative accumulation of WCMV CP of AMV and WCMV co-infection. AMV CP (AMV:WCMV), relative accumulation of AMV CP of AMV and WCMV co-infection. (**A**) The 9 dpi samples were normalized to 1.0 for each treatment. The relative accumulation of AMV CP and WCMV CP at 15 dpi, 21 dpi, and 27 dpi was calculated. The different lowercase letters indicate significant differences at the 0.05 level between AMV and WCMV co-infections and AMV or WCMV single infections at different times in the same treatment. (**B**) The WCMV single-infection samples were normalized to 1.0 each time (dpi). The relative accumulation of AMV CP and WCMV CP (AMV:WCMV) at 9 dpi, 15 dpi, 21 dpi, and 27 dpi was calculated. The different capital letters indicate significant differences at the 0.01 level between AMV and WCMV co-infection and AMV or WCMV single infection at different treatments at the same time.

Meanwhile, the RT-qPCR determination results showed that the relative accumulation of AMV CP and WCMV CP in *N. benthamiana* co-infections was significantly higher than that in AMV and WCMV single infections. When 9 dpi samples were normalized to 1.00 for each treatment, the relative accumulation of AMV CP and WCMV CP reached a higher level at 21 dpi (Figure 4A). When the relative accumulation of WCMV CP in the single infection was normalized to 1.00, the relative accumulation of AMV CP and WCMV CP in co-infection was 15.44-fold and 10.04-fold greater than the respective values for single virus infection, respectively (Figure 4B). Generally, the concentrations of AMV and WCMV and the relative accumulation of AMV CP and WCMV CP were increased in the co-infection, indicating a synergistic effect between AMV and WCMV.

### 3.3. Changes in the Contents of Photosynthetic Pigments in N. benthamiana after AMV and WCMV Co-Infection

**Figure 5:**
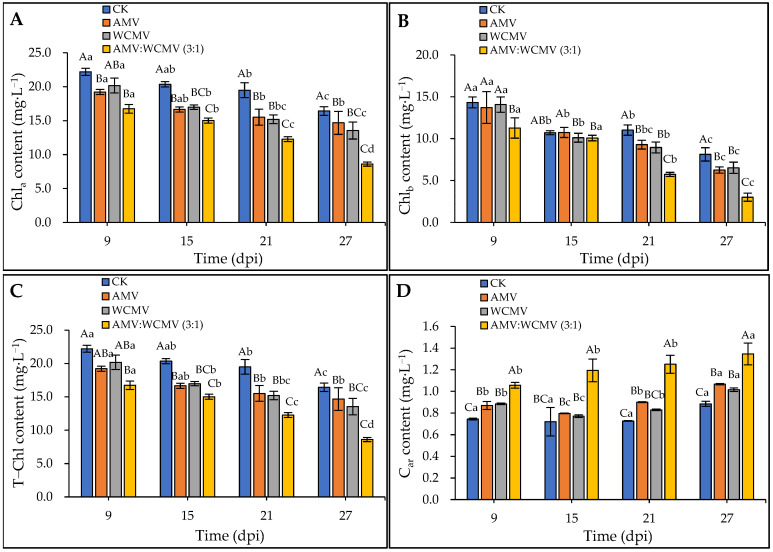
Changes in photosynthetic pigment content of *N. benthamiana* after AMV and WCMV co-infection. (**A**) The changes in Ch1_a_ content in *N. benthamiana*. (**B**) The changes in Ch1_b_ content in *N. benthamiana*. (**C**) The changes in T-Ch1 content in *N. benthamiana*. (**D**) The changes in C_ar_ content in *N. benthamiana*. The different capital letters indicate significant differences at the 0.01 level between AMV and WCMV co-infection and AMV or WCMV single infection in different treatments at the same time, and the different lowercase letters indicate significant differences at the 0.05 level between AMV and WCMV co-infection and AMV or WCMV single infection at different times in the same treatments.

The AMV and WCMV co-infections significantly influenced the photosynthetic pigments in *N. benthamiana* leaves. Specifically, the Ch1_a_, Ch1_b_, and T-Ch1 contents exhibited a decreasing trend over time. In addition, AMV and WCMV co-infection significantly reduced the *N. benthamiana* Ch1_a_, Ch1_b_, and T-Ch1 contents compared to AMV or WCMV single infections and CK (Figure 5). At 27 dpi, the Ch1_a_ content was 8.60 mg·L^−1^ in *N. benthamiana* after co-infection with AMV and WCMV and reduced by 21.88, 10.51, and 30.84% compared to AMV (14.68 mg·L^−1^) and WCMV (13.54 mg·L^−1^) single infections and CK (16.42 mg·L^−1^), respectively (Figure 5A). On the other hand, there was no significant difference in the Ch1_b_ content in *N. benthamiana* leaves co-infected with AMV and WCMV compared to WCMV single infection and CK at 15 dpi. At 27 dpi, the Ch1_b_ content was 3.02 mg·L^−1^ in *N. benthamiana* after being co-infected with AMV and WCMV and reduced by 51.81%, 53.81%, and 62.92% compared to AMV (6.26 mg·L^−1^) and WCMV (6.53 mg·L^−1^) single infections and CK (8.14 mg·L^−1^), respectively (Figure 5B). Additionally, the T-Ch1 content in the leaves co-infected with AMV and WCMV reached its lowest value of 11.62 mg·L^−1^, which was 51.81%, 53.81%, and 62.92% lower than in the *N. benthamiana* with AMV (6.26 mg·L^−1^) and WCMV (6.53 mg·L^−1^) single infections and CK (8.14 mg·L^−1^), respectively (Figure 5C). On the contrary, the C_ar_ content exhibited an increasing trend over time. The *N. benthamiana* with AMV and WCMV co-infection had a significantly higher C_ar_ content than AMV and WCMV single infections and CK. At 27 dpi, the C_ar_ content was 1.35 mg·L^−1^ in *N. benthamiana* after co-infection with AMV and WCMV and reduced by 20.71%, 24.54%, and 34.30% compared to AMV (1.07 mg·L^−1^) and WCMV (1.02 mg·L^−1^) single infections and CK (0.88 mg·L^−1^), respectively (Figure 5D).

### 3.4. The Changes in Gas Exchange During Photosynthesis in N. benthamiana after AMV and WCMV Co-Infection

**Figure 6:**
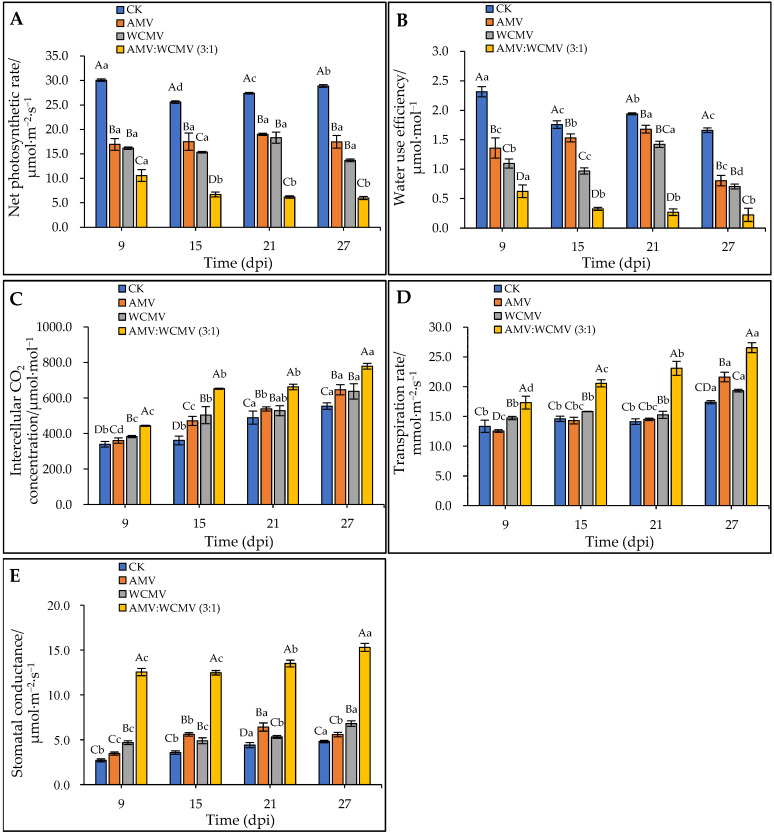
Effect on parameters for gas exchange in the photosynthesis of *N. benthamiana* leaves after AMV and WCMV co-infection. (**A**) Net photosynthetic rate (*P*_n_). (**B**) Water use efficiency (WUE). (**C**) Intercellular CO_2_ concentration (*C*_i_). (**D**) Transpiration rate (*T*_r_). (**E**) Stomatal conductance (*G*_s_). The different capital letters indicate significant differences at the 0.01 level between AMV and WCMV co-infection and AMV or WCMV single infection in different treatments at the same time, and the different lowercase letters indicate significant differences at the 0.05 level between AMV and WCMV co-infection and AMV or WCMV single infection at different times in the same treatments.

To verify the impact of AMV and WCMV on photosynthesis, the change in gas exchange was measured after AMV and WCMV co-infection in *N. benthamiana*. The AMV and WCMV co-infection of *N. benthamiana* had a decreasing trend in *P*_n_ and WUE contents with the increase in time, whereas at the same time, the *P*_n_ and WUE contents of the AMV and WCMV co-infection were significantly lower than those of the AMV and WCMV single infection, or CK. Meanwhile, the *P*_n_ and WUE contents of the two single-infection viruses were significantly lower than those of the CK. At 27 dpi, the content of *P*_n_ was 5.96 μmol·m^−2^·s^−1^, which was 65.84%, 56.37%, and 79.36% lower than those of AMV (17.45 μmol·m^−2^·s^−1^) and WCMV (13.66 μmol·m^−2^·s^−1^) single infections and CK (28.88 μmol·m^−2^·s^−1^), respectively (Figure 6A). The WUE content was 0.22 μmol·mol^−1^, which was 72.06%, 68.21%, and 86.48% lower than those of AMV (0.80 μmol·mol^−1^) and WCMV (0.71 μmol·mol^−1^) single infections and CK (1.66 μmol·mol^−1^), respectively (Figure 6B).

In contrast, AMV and WCMV co-infection in *N. benthamiana* had an increasing trend of *C*_i_, *T*_r_, and *G*_s_ contents with the increase in time, whereas at the same time, the *C*_i_, *T*_r_, and *G*_s_ contents of AMV and WCMV co-infection were significantly higher than those of AMV and WCMV single infection, or CK. Meanwhile, the *C*_i_, *T*_r_, and *G*_s_ contents of the two single-infection viruses were significantly higher than those of the CK. By 27 dpi, the content of *C*_i_ was 778.77 μmol·mol^−1^, which was 16.70%, 18.20%, and 28.81% lower than those of AMV (646.40 μmol·mol^−1^) and WCMV (637.07 μmol·mol^−1^) single infections and CK (554.41 μmol·mol^−1^) (Figure 6C). The content of *T*_r_ was 26.54 mmol·m^−2^·s^−1^, which was 18.54%, 27.18%, and 34.46% lower than those of AMV (21.62 mmol·m^−2^·s^−1^) and WCMV (19.33 mmol·m^−2^·s^−1^) single infections and CK (17.40 mmol·m^−2^·s^−1^) (Figure 6D). The content of *G*_s_ was 15.31 μmol·m^−2^·s^−1^, which was 63.55%, 55.58%, and 68.62% lower than those of AMV (5.58 μmol·m^−2^·s^−1^) and WCMV (6.80 μmol·m^−2^·s^−1^) single infections and CK (4.80 μmol·m^−2^·s^−1^), respectively (Figure 6E). Thus, these results indicate that AMV and WCMV co-infection changes the content of the gas exchange parameter, making *N. benthamiana* more susceptible to disease.

### 3.5. The Changes in Chlorophyll Fluorescence Parameters in N. benthamiana after AMV and WCMV Co-Infection

**Figure 7:**
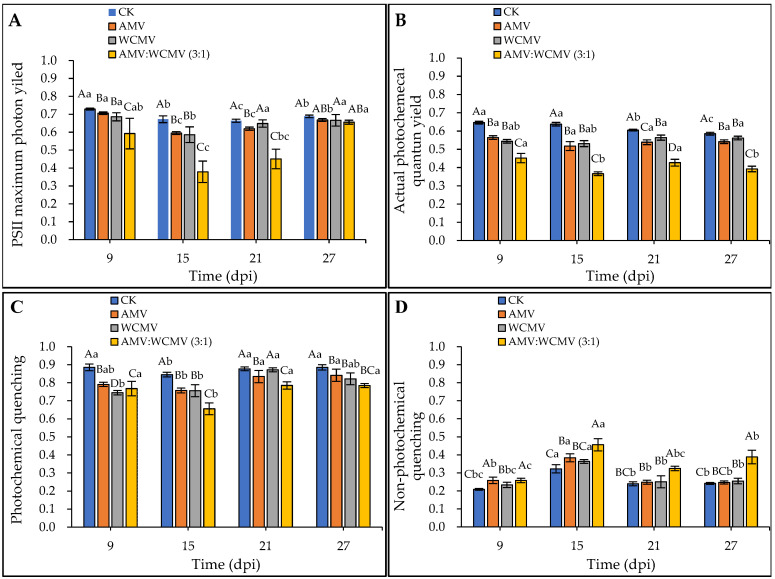
Dynamics of chlorophyll fluorescence parameters in *N. benthamiana* after AMV and WCMV co-infection. (**A**) PSII maximum photon yield (*F_v_*/*F_m_*). (**B**) Actual photochemical quantum yield (Φ_PSII_). (**C**) Photochemical quenching (*q*P). (**D**) Non-photochemical quenching (*q*N). The different capital letters indicate significant differences at the 0.01 level between AMV and WCMV co-infection and AMV or WCMV single infection in different treatments at the same time, and the different lowercase letters indicate significant differences at the 0.05 level between AMV and WCMV co-infection and AMV or WCMV single infection at different times in the same treatments.

The effect of the AMV and WCMV co-infection on chlorophyll fluorescence parameters in *N. benthamiana* was determined. The *F_v_/F_m_*, Φ_PSII_, and *q*P of AMV and WCMV co-infection were significantly lower than those of AMV and WCMV single infection and CK, exhibiting an initial decline followed by a subsequent increase at a different time. At 15 dpi, the *F_v_/F_m_*, Φ_PSII_, and *q*P reduced the value to the minimum in *N. benthamiana* of AMV and WCMV co-infection, and the *F_v_/F_m_* values were 0.38 and significantly reduced by 36.29%, 35.32%, and 43.64% compared to the values recorded in *N. benthamiana* with AMV (0.60) and WCMV (0.59) single infection and CK (0.67), respectively (Figure 7A). The Φ_PSII_ values were lowest (0.37), which were 29.31%, 31.06%, and 42.57% lower than those of AMV (0.52), WCMV (0.53), and CK (0.64), respectively (Figure 7B). The *q*P value was 0.66, which was 13.39%, 13.25%, and 22.45% lower than those of AMV (0.76) and WCMV (0.76) single infections and CK (0.85), respectively (Figure 7C).

Additionally, AMV and WCMV co-infection in *N. benthamiana* had a rising and then decreasing trend in *q*N values with the increase in time; it rose to its maximum at 15 dpi. The *q*N values of AMV and WCMV co-infection were significantly higher than those of AMV and WCMV single infection and CK at the same time. Meanwhile, the *q*N values of the two viruses per single infection were significantly higher than those of the CK. At 15 dpi, the *q*N was 0.46, which was 15.87%, 20.31%, and 29.29% lower than those of AMV (0.38) and WCMV (0.36) single inoculations and CK (0.32), respectively (Figure 7D). These results also indicate that AMV and WCMV co-infection changes the chlorophyll fluorescence parameters, destroys the photoprotective mechanism, and increases the degree of disease in *N. benthamiana*.

## 4. Discussion

### 4.1. Synergistic Effect of AMV and WCMV Co-Infection

At present, although the mechanisms of pathogenicity of AMV and WCMV in single infections of different plants have been thoroughly studied [26,27], the interaction between AMV and WCMV co-infection in plants is less well understood. Our previous study revealed that clover virus disease caused by AMV and WCMV is widespread, there is a co-infection phenomenon that can lead to substantial losses, and the relative contents of the two viruses were significantly increased in co-infected white clover [19]. Several studies have revealed that chloroplasts are the most susceptible organelles to viral infection and the first organelles attacked by most plant viruses [28,29,30]. Here, we have demonstrated that AMV and WCMV co-infection results in a synergistic interaction, leading to more severe disease symptoms of mosaic, mottling, and dwarfing of the whole *N. benthamiana* than AMV and WCMV single infections. Meanwhile, the changes in chloroplast structure include the contents dissolving, with the cell membrane structure being disorganized, and even the chloroplast structure disintegrating [31]. In addition, the relative accumulation of AMV CP and WCMV CP was significantly increased by 15.44-fold and 10.04-fold upon co-infection compared to that with a single virus infection at 21 dpi. It is worth noting why the concentration of AMV and WCMV and the relative accumulation of AMV CP and WCMV CP reached their max at 21 dpi. This may be due to the fact that after the virus invades the host chloroplast, it causes corresponding damage to the host cell while providing raw materials for its replication [19]. At 21 dpi, the host cell damage rate reaches its maximum, and the virus cannot be provided with raw materials for replication, resulting in a gradual decrease in the virus’s replication rate [32,33]. The specific reasons remain to be further studied.

### 4.2. Effect on Photosynthetic Characteristics of N. benthamiana after AMV and WCMV Co-Infection

The effect of virus infection on host photosynthesis has been widely reported [34]. It has been suggested that altered photosynthesis is a common and conservative strategy for viral pathogenesis [35,36]. Plant viruses impede the normal physiological metabolism of the host after infestation, increase the activity of chlorophyll catabolic enzymes, and decrease the chlorophyll content [29,37,38]. In addition, viral infections affecting the electron transfer activity within plant chloroplasts significantly restrain the absorption and capture capacity of the light system [39]. This means that chloroplast–virus interactions include decreases in chlorophyll pigments, changes in photosynthetic gas exchange parameters, and chlorophyll fluorescence [40,41].

We therefore investigated the effect of AMV and WCMV co-infection on the photosynthetic properties of *N. benthamiana* leaves. This result indicated that the Ch1_a_, Ch1_b_, and T-Ch1 contents of the AMV and WCMV co-infection were significantly lower than those of the AMV and WCMV single infection, or CK; the contents of C_ar_ were increased. Meanwhile, connected with the timing of virus inoculation, the Ch1_a_, Ch1_b_, and T-Ch1 content decreased gradually with increasing inoculation time and car content, on the contrary [42]. Moreover, under AMV and WCMV co-infection, the gas exchange during the photosynthesis effects of *N. benthamiana* were more severe, and the *P*_n_ and WUE content significantly reduced compared to AMV and WCMV single inoculations and CK. With increasing inoculation time, the *C*_i_, *T*_r_, and *G*_s_ content has been increasing with decreasing inoculation time. With the enhancement in inoculation time over a range, the PS II photosystem potential activity was significantly lower, with lower *F_v_/F_m_*, Φ_PSII_, and *q*P values than the AMV and WCMV single inoculations and CK, and higher *q*N values than these three treatments [43,44]. It was further confirmed that the disease symptoms of *N. benthamiana* after AMV and WCMV co-infection were more serious than those of AMV and WCMV single infection, with more serious harm to the host and a stronger effect of viral infection on the structure of the chloroplasts, which leads to a reduction in the chlorophyll content and a change in the photosynthetic activity of the host [45].

In addition, we inoculated a mixture of AMV and WCMV (3:1) on different host plants of the Leguminosae, Solanaceae, Poaceae, Cucurbitaceae, Asteraceae, Brassicaceae, and Chenopodiaceae, which are different host plants. It was found that under these conditions, the co-infection of these two viruses would aggravate the disease symptoms of the host plants, and the damage was higher than that caused by the single infection of the viruses. At the same time, *Nicotiana tabacum*, *S. tuberosum*, and *C. annuum* in the Solanaceae family are the main cash crops in China, which are widely planted and are also the main hosts of AMV and WCMV infection, especially *N. tabacum*, which is planted in a large area in rural areas. We have conducted research on the photosynthetic characteristics of *N. tabacum* after inoculation with AMV and WCMV, and the results showed that the impact of the co-infection with AMV and WCMV on *N. tabacum* was consistent with that on *N. benthamiana*. This indicates that the synergistic effect among viruses significantly inhibited the normal conduct of photosynthesis and reduced the photosynthetic efficiency of the plant compared with a single infection, affecting the physiological functions and growth of the hosts [45,46]. Thus, these findings will provide a more comprehensive understanding of the inhibitory synthesis of photosynthetic pigments and chloroplast photosynthesis with virus-co-infected plants [47].

Our results provide the first evidence that the combined infection of AMV and WCMV aggravated the morbidity symptoms of *N. benthamiana*, severely damaged the chloroplast morphology results, and interfered with the normal conduct of photosynthesis. There was a synergistic effect between AMV and WCMV, with WCMV as the synergistic virus and AMV as the passive synergistic virus.

## Figures and Tables

**Table 1:** Standard curve formulas for target gene and internal gene.

Primer Name	Formula	R^2^	Amplification Efficiency (*E*)
AMV CP	y = −3.3586x + 33.941	R^2^ = 0.9939	0.9849
WCMV CP	y = −3.3452x + 32.064	R^2^ = 0.9898	0.9904
25S rRNA	y = −3.3252x + 27.819	R^2^ = 0.9882	0.9986
β-Actin	y = −3.2386x + 26.667	R^2^ = 0.9885	1.0360

## Data Availability

The raw data supporting the conclusions of this article will be made available by the authors without undue reservation.
